# Evidence for a functional role of *Start*, a long noncoding RNA, in mouse spermatocytes

**DOI:** 10.1371/journal.pone.0273279

**Published:** 2022-08-25

**Authors:** Kai Otsuka, Hong Yang, Shin Matsubara, Akira Shiraishi, Misuzu Kurihara, Honoo Satake, Atsushi P. Kimura

**Affiliations:** 1 Graduate School of Life Science, Hokkaido University, Sapporo, Japan; 2 Department of Microbiology and Molecular Genetics, University of California, Davis, California United States of America; 3 Bioorganic Research Institute, Suntory Foundation for Life Sciences, Kyoto, Japan; 4 RNA Biology Laboratory, Faculty of Pharmaceutical Sciences, Hokkaido University, Sapporo, Japan; 5 Department of Biological Sciences, Faculty of Science, Hokkaido University, Sapporo, Japan; China University of Science and Technology, CHINA

## Abstract

A mouse testis-specific long noncoding RNA (lncRNA), *Start*, is localized in the cytosol of Leydig cells and in the nucleus of pachytene spermatocytes. We previously showed that *Start* regulates steroidogenesis through controlling the expression of *Star* and *Hsd3b1* genes in Leydig cells, but its function in germ cells was not known. Here we verified that a spermatocyte-specific protease gene, *Prss43/Tessp-3*, was downregulated in *Start*-knockout testes. To investigate the transcriptional regulatory activity of *Start* in spermatocytes, we first performed a series of reporter gene assays using a thymidine kinase promoter in spermatocyte-derived GC-2spd(ts) cells. A 5.4-kb genome sequence encompassing *Start* exhibited enhancer activity for this promoter, and the activity was decreased by knockdown of *Start*. Deletion of the *Start* promoter and replacement of the *Start* sequence abolished the enhancer activity and, consistently, the activity was detected in further experiments only when *Start* was actively transcribed. We then examined whether the *Prss43/Tessp-3* gene could be a target of *Start*. A reporter gene assay demonstrated that the 5.4-kb sequence exhibited enhancer activity for a *Prss43/Tessp-3* promoter in GC-2spd(ts) cells and that the activity was significantly decreased by knockdown of *Start*. These results suggest that *Start* functions in transcriptional activation of the *Prss43/Tessp-3* gene in spermatocytes. Given that *Start* is presumed to regulate steroidogenic genes at the posttranscriptional level in Leydig cells, the function in spermatocytes is a novel role of *Start*. These findings provide an insight into multifunctionality of lncRNAs in the testis.

## Introduction

Long noncoding RNAs (lncRNAs) are a class of noncoding RNAs that are longer than 200 nucleotides and play important roles in various biological events [1, 2]. In mammals, the testis is known to express more lncRNAs than other tissues [3, 4], and transcriptome studies have revealed dynamic change of lncRNA expression during spermatogenesis [5–7]. These findings suggest biological significance of lncRNAs in the testis. Indeed, knockout (KO) mouse models of several testis-specific lncRNAs showed defects in the production or quality of sperm [8–10], and *in vivo* knockdown of lncRNAs in the testis resulted in obvious impairment of spermatogenesis [11, 12]. However, only a few lncRNAs have been functionally assessed.

Interestingly, some lncRNAs were found to have multiple roles [13–16]. For example, treRNA/ncRNA-a7 enhances gene transcription in the nucleus of some human cell lines, while it suppresses translation in the cytosol of breast cancer cells [17, 18]. *H19* plays a role in genomic imprinting in the nucleus of neonatal liver cells and acts as a decoy against miRNAs in ovarian and kidney cells [19, 20]. *Gas5* interacts with miRNAs in the cytosol of gastric cancer cells, while it is a component of nuclear structures in fibroblasts and antagonizes the glucocorticoid receptor in the nucleus of Hela cells [21–23]. These findings indicate that multifunctional lncRNAs play different roles according to cell types or subcellular localization. Since there are many lncRNAs expressed in multiple tissues or localized in different subcellular compartments [24–26], other lncRNAs are highly likely to be multifunctional.

**Fig 1:**
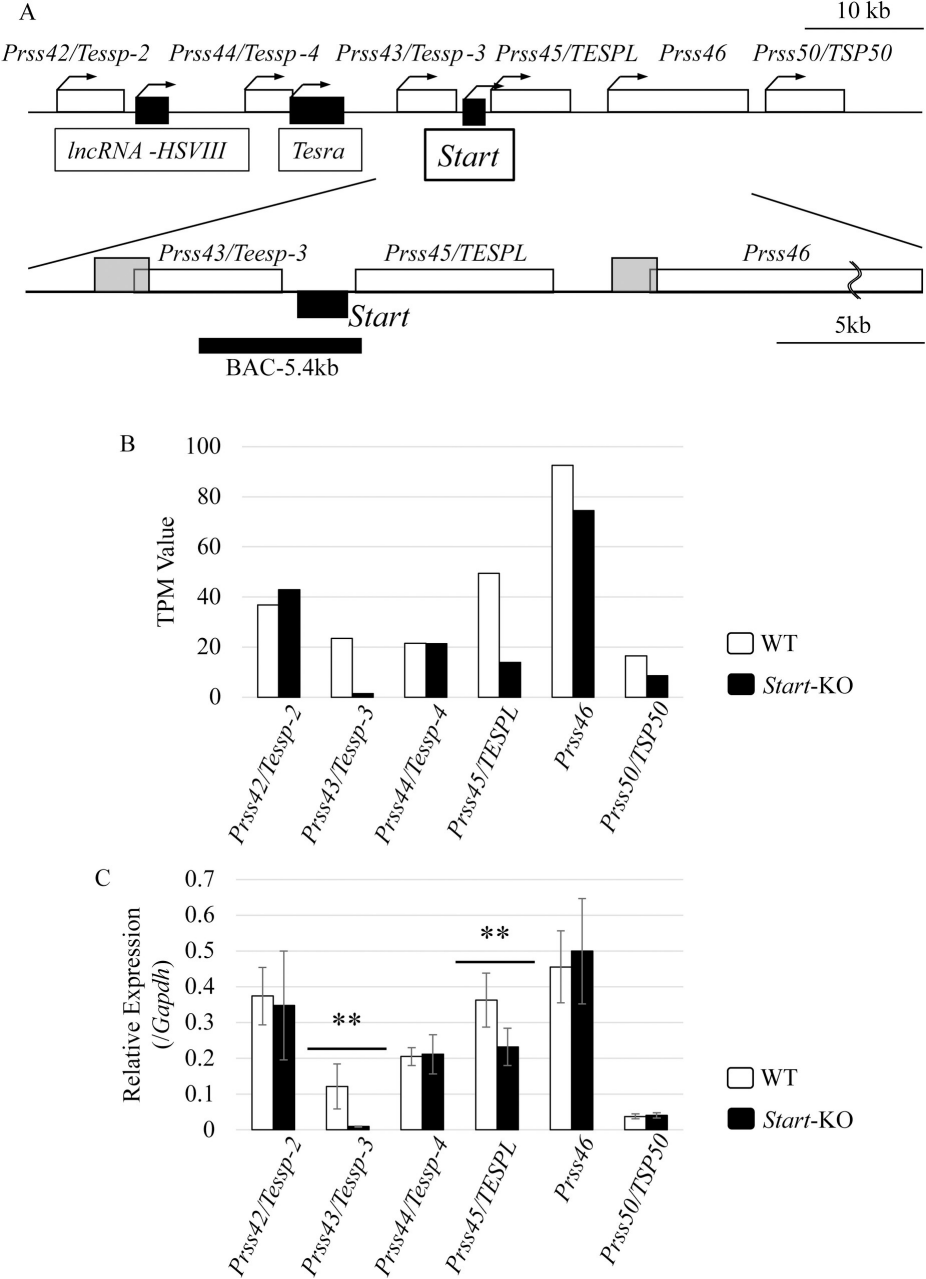
Expression of *Prss/Tessp* genes in *Start*-KO and wild-type testes. (A) A schematic drawing of a 75-kb genomic region at mouse chromosome 9 (9: 110,625,000–110,700,000). Six white boxes specify protein-coding *Prss/Tessp* genes, whereas three black boxes are lncRNAs. Bent arrows indicate the transcriptional direction of each gene. An enlarged drawing at the bottom indicates positions of promoters and a BAC-derived sequence used in this study. Two grey boxes at the left-end of *Prss43/Tessp-3* and *Prss46* genes are regions of putative promoters, and a bold black bar is a region corresponding to a BAC-derived sequence that encompasses the full length of the *Start* sequence. (B) Expression of *Prss/Tessp* genes in the testis at 2.5 months determined by RNA-seq analysis. Total RNAs were purified from 2.5-month-old wild-type and *Start*-KO testes and used for RNA-seq analysis. The TPM values of *Prss42/Tessp-2*, *Prss43/Tessp-3*, *Prss44/Tessp-4*, *Prss45/TESPL*, *Prss46*, and *Prss50/Tsp50* in *Start*-KO and wild-type testes were calculated. White bars represent the data from a wild-type testis, and black bars represent those from a *Start-*KO testis. RNA-seq was done with one set of littermates. (C) Expression of the *Prss/Tessp* genes in the testis at 2.5 months determined by qRT-PCR. Total RNAs were purified from 2.5-month-old wild-type and *Start*-KO testes and used for qRT-PCR. The *Gapdh* gene was used as an internal control to normalize the expression level of each gene. Data are presented as means ± S.D. from four sets of wild-type and KO littermates. Statistical significance was analyzed by Student’s t-test. **P < 0.01.

We have focused on lncRNAs that are transcribed at the mouse *Prss/Tessp* locus, which encodes six testis-specific protease genes and three lncRNAs [27–29] (Fig 1A). *Start*, an lncRNA at this locus, is expressed in both Leydig cells and germ cells. In our previous study, we verified that *Start* regulates the production of testosterone via activation of two steroidogenic genes, *Star* and *Hsd3b1*, in Leydig cells [30]. Localization of *Start* in the cytosol of Leydig cells led to the presumption that the steroidogenic genes are regulated at the posttranscriptional level by *Start*. In contrast, in pachytene spermatocytes, *Start* is localized in the nucleus [30]. These results suggest that *Start* functions at the transcriptional level.

In this study, we investigated the role of *Start* in spermatocytes and revealed that *Start* transcript plays a role in transcriptional activation of a neighboring spermatocyte-specific gene, *Prss43/Tessp-3*. This strongly suggests that *Start* is a multifunctional lncRNA that regulates steroidogenesis in Leydig cells and activates the transcription of a protease gene in spermatocytes.

## Materials and methods

### Animals

The experimental procedures used in this study were approved by the Institutional Animal Use and Care Committee at Hokkaido University. *Start*-KO mice were generated as previously described [30]. The mice were maintained on 14 hr light/10 hr dark cycles at 25℃ and given food and water ad libitum. In the analysis of *Start*-KO mice, we used male mice at 64–83 days postpartum.

### RNA-sequencing (RNA-seq) analysis

RNA-sequencing (RNA-seq) data (SRR12700726 and SRR12700727) were analyzed as previously described [30]. The expression level of each gene was calculated as gene-specific transcript per million mapped reads (TPM).

### Quantitative reverse transcription-polymerase chain reaction (qRT-PCR) analysis

Total RNAs were extracted by ISOGEN (Nippon Gene, Tokyo, Japan) and ISOGEN II (Nippon Gene) for testes and cultured cells, respectively, according to the manufacturer’s instructions. After treatment with TurboDNase (Thermo Fisher Scientific, Waltham, MA, USA), the RNAs were reverse-transcribed into cDNAs with the oligo(dT) primer using Superscript III (Thermo Fisher Scientific), according to the manufacturer’s instructions. PCR was performed by using SYBR Green PCR Master Mix (Thermo Fisher Scientific). The 7300 real-time PCR system (Applied Biosystems, Foster City, CA, USA) was used. The relative expression levels were normalized to an endogenous *Gapdh* mRNA. Primer sequences are shown in Table 1.

**Table 1:** Oligo DNA sequences used in this study.

Name	Forward	Reverse
[qRT-PCR]		
*Prss42/Tessp-2*	ATGCATGTCTGTGGAGGTTC	CTGAAAGTGTGACCCTGGTC
*Prss43/Tessp-3*	GGTCTGCAAGACTCAGGACA	AGAGGACAGGGACTCCATTG
*Prss44/Tessp-4*	CAAGGACATCATGGGGAATA	CTACCTGCACCCACGTTTTA
*Prss45/TESPL*	GGGAGGACTTGTGCTATGGA	ATGAGAATACCCCAGCCAGA
*Prss46*	TGGTTTGCCAGATGAACAAG	TGGTGTAGACGCTTGGGAAT
*Prss50/Tsp50*	CCACCGAACTCACAGACCAT	GTTGCTGGAATGAACCGTCT
*Gapdh*	TGCACCACCAACTGCTTAGC	GGCATGGACTGTGGTCATGAG
*Start*	CCCACTCTTAGCCTCATGGT	CCATCACCCAGCCTGTTCGTT
[shRNA]		
shRNA-v1	GATCCGGACAATCAGTTGTTCCTTTGCTGTGAAGCCACAGATGGGCAAAGGAACAACTGATTGTCCTTTTTTA	AGCTTAAAAAAGGACAATCTGTTGTTCCTTTGCCCATCTGTGGCTTCACAGCAAAGGAACAACTGATTGTCCG
shRNA-v2	GATCCGGATGAAGATTTCTAGCAAGTCTGTGAAGCCACAGATGGGACTTGCTAGAAATCTAACATCCTTTTTTA	AGCTTAAAAAAGGATGAAGATTTCTAGCAAGTCCCATCTGTGGCTTCACAGACTTGCTADAAATGTTCATCC
[RT-PCR]		
*Start*	CCCACTCTTAGCCTCATGGT	ATCGCATATCGAGGCAAGCA
*Gapdh*	CATGACCACAGTCCATGCCATC	TAGCCCAAGATGCCCTTCAGTG
[Cloning]		
*Prss43/Tessp-3* Promoter	GGTACTCATGCGCCTCGAAA	GGTCCTGAGTTAAGCCCGAA
*Prss46* Promoter	GCTCCTATCCTCCTGTCCCT	TCCACCGCCTTCCCATTTAC

### Plasmid construction

The thymidine kinase (TK) promoter was obtained as a 750-bp fragment from a pEBMulti-Neo vector (Fujifilm Wako, Osaka, Japan) by digestion with *Bgl*II and *Hin*dIII. This 750-bp promoter sequence was inserted at the *Bgl*II/*Hin*dIII site of a pGL3-Basic vector (Promega, Madison, WI, USA). The resulting construct was denoted as “Control”.

To obtain *Start* and its flanking sequence, a bacterial artificial chromosome clone encompassing the *Prss/Tessp* gene cluster (B6Ng01‐306O15, RIKEN Bioresource Center, Tsukuba, Japan) was digested with *Sac*II, and a 5.4-kb fragment was blunted and subcloned into the *Eco*RV site of a pBluescript II KS(+) vector. This “pBlue-BAC-5.4kb” plasmid was then digested with *Not*I and *Sal*I, and the 5.4-kb fragment was blunted and inserted at the blunted *Bam*HI site of “Control”. The resulting construct was denoted as “TK-BAC”.

“TK-BAC” was digested with *Apa*I to separate the 10.9-kb sequence into 1.6-kb and 9.3-kb fragments. The 1.6-kb fragment was further digested with *Eco*RV, and a longer 1.3-kb fragment was collected. Then the 9.3-kb and 1.3-kb fragments were blunted and ligated to each other, resulting in the generation of “ΔProm” that lacked an approximately 350-bp region around the transcriptional start site of *Start* (S1A Fig). For construction of “λEco” and “λApa”, “TK-BAC” was digested with *Eco*RV/*Sal*I and *Apa*I/*Sal*I, respectively, and blunted. Then, a 2.3-kb λHindIII fragment was blunted and ligated to each vector. This resulted in the completion of “λEco” and “λApa” (S1B and S1C Fig).

The full-length sequence of *Start* was subcloned into a pBluescript II KS(+) vector as previously described [30]. The *Start* sequence was digested out with *Eco*RI and *Hin*dIII, blunted, and inserted at the blunted *Bam*HI site of “Control” in different directions. The resulting constructs were denoted as “Start-Forward” and “Start-Reverse”.

To generate constructs containing a half of the 5.4-kb BAC fragment, “pBlue-BAC-5.4kb” was digested with *Apa*I, and 1.6-kb and 4.0-kb fragments were collected and ligated to each other. The resulting plasmid was digested with *Not*I and *Sal*I, blunted, and inserted at the blunted *BamH*I site of “Control”. The resulting construct that contained the second half of the 5.4-kb fragment was denoted as “TK-BAC-SH” (S1D Fig). On the other hand, a 2.7-kb fragment was collected after digestion of “pBlue-BAC-5.4kb” with *Apa*I, blunted, and inserted at the blunted *Bam*HI site of “Control”. The resulting construct is “TK-BAC-FH”, which contained the first half of the 5.4-kb fragment (S1E Fig).

The promoter sequences of *Prss43*/*Tessp-3* and *Prss46* genes were amplified by PCR with pairs of primers listed in Table 1. Each PCR product was inserted at the *Sma*I site of a pGL3-Basic vector (Promega). The resulting constructs were denoted as “Pr43-Cont” and “Pr46-Cont”. A 5.4-kb BAC fragment was obtained by digestion of “pBlue-BAC-5.4kb” with *Not*I and *Sal*I, blunted, and inserted at the blunted *Bam*HI site. The resulting constructs were denoted as “Pr43-BAC” and “Pr46-BAC”. The length of each promoter (2.3 kb for *Prss43/Tessp-3* and 1.0 kb for *Prss46*) was determined according to previous studies [31, 32].

For knockdown (KD) experiments, a pBAsi-mU6 Neo vector (Takara, Shiga, Japan) was modified to have hygromycin resistance. The SV40 promoter and the hygromycin resistance gene were obtained by PCR using a Glo Sensor-22F vector (Promega) and ligated to the blunted *Eco*RI site of pBAsi-mU6 Neo. Two shRNAs for *Start* transcript were designed by the web tool BLOCK-iT RNAi Designer (https://rnaidesigner.thermofisher.com). Sense and antisense oligo DNAs of the designed sequences (Table 1) were annealed and inserted at the *Bam*HI/*Hin*dIII site of the modified vector (“shRNA-v1” and “shRNA-v2”).

### Cell culture and transfection

GC-2spd(ts) cells (CRL-2196) were obtained from American Type Culture Collection and were cultured in DMEM (Fujifilm Wako) containing 10% fetal bovine serum, 100 U/ml penicillin, 100 μg/ml streptomycin, and 292 μg/ml L-glutamine (Nacalai tesque, Kyoto, Japan). For transient transfection, 2.0×10^4^ cells were seeded in each well of a 24-well plate one day before transfection. Five hundred nanograms of DNA constructs was used for transfection with GeneJuice (Merck, Darmstadt, Germany) according to the manufacturer’s protocol. To establish GC-2spd(ts) cells that were stably transfected with luciferase constructs, a pKO-SelectPuro V810 vector (Lexicon Genetics, The Woodlands, TX, USA) was co-transfected, and the selection was done with 3 μg/ml puromycin for 2 weeks. shRNA-v1 and shRNA-v2 were transfected into the stable cells and the cells were treated with 300–500 μg/ml hygromycin for 5–10 days.

### Reporter gene assay

All constructs for the reporter gene assay contained the firefly luciferase gene. For a transient assay, the pRL‐CMV vector (Promega), in which the *Renilla* luciferase gene was driven by the CMV promoter, was co-transfected with experimental constructs. Luciferase activity was measured 2 days after transfection with a Dual-Luciferase Reporter Assay System (Promega) using Lumat LB9507 (Berthold, Bad Wildbad, Germany). The value of firefly luciferase was normalized to that of *Renilla* luciferase for adjusting transfection efficiency in each trial. For stable cells, the value of firefly luciferase was normalized to the protein amount that was determined by using a BCA Protein Assay Kit (Thermo Fisher Scientific).

### RT-PCR analysis

cDNAs were synthesized as described above using the oligo(dT) primer. PCR was performed using ExTaq polymerase (Takara) or KOD Fx Neo (Toyobo, Osaka, Japan), and the products were analyzed by electrophoresis on agarose gels. Primer sequences are shown in Table 1.

### Statistical analysis

Results are presented as the mean value ± standard deviation (S.D.) of at least three independent experiments. The data were statistically analyzed by Student’s t-test or one-way analysis of variance (ANOVA) followed by Dunnett’s test or post-hoc Tukey HSD test. A P value less than 0.05 was considered statistically significant. All statistical calculations were done by R software (Ver. 3.5.0; https://cran.ism.ac.jp/bin/windows/).

## Results

### *Prss43/Tessp-3 and Prss45/TESPL* genes were downregulated in *Start-*KO testes

We previously generated *Start*-KO mice and performed RNA-seq analysis with whole testes of 2.5-month-old wild-type and *Start*-KO mice [30]. As a result of calculation of TPM values, expression of two neighboring genes of *Start*, *Prss43/Tessp-3* and *Prss45/TESPL*, was decreased to 6% and 28%, respectively, in *Start*-KO testes (Fig 1B). For further confirmation, we performed qRT-PCR using four sets of adult *Start*-KO and wild-type testes and revealed that *Prss43/Tessp-3* and *Prss45/TESPL* genes were significantly downregulated to 8% and 64%, respectively, in *Start*-KO testes (Fig 1C). The two genes were transcriptionally activated in primary spermatocytes [27, 33] and *Start* is localized in the nucleus at this meiotic stage, implying a role of *Start* in transcriptional activation in spermatocytes. In this study, we focused on regulation of the *Prss43/Tessp-3* gene, which was more prominently affected by *Start*-KO.

### Activation of a thymidine kinase promoter by the *Start* transcript

**Fig 2:**
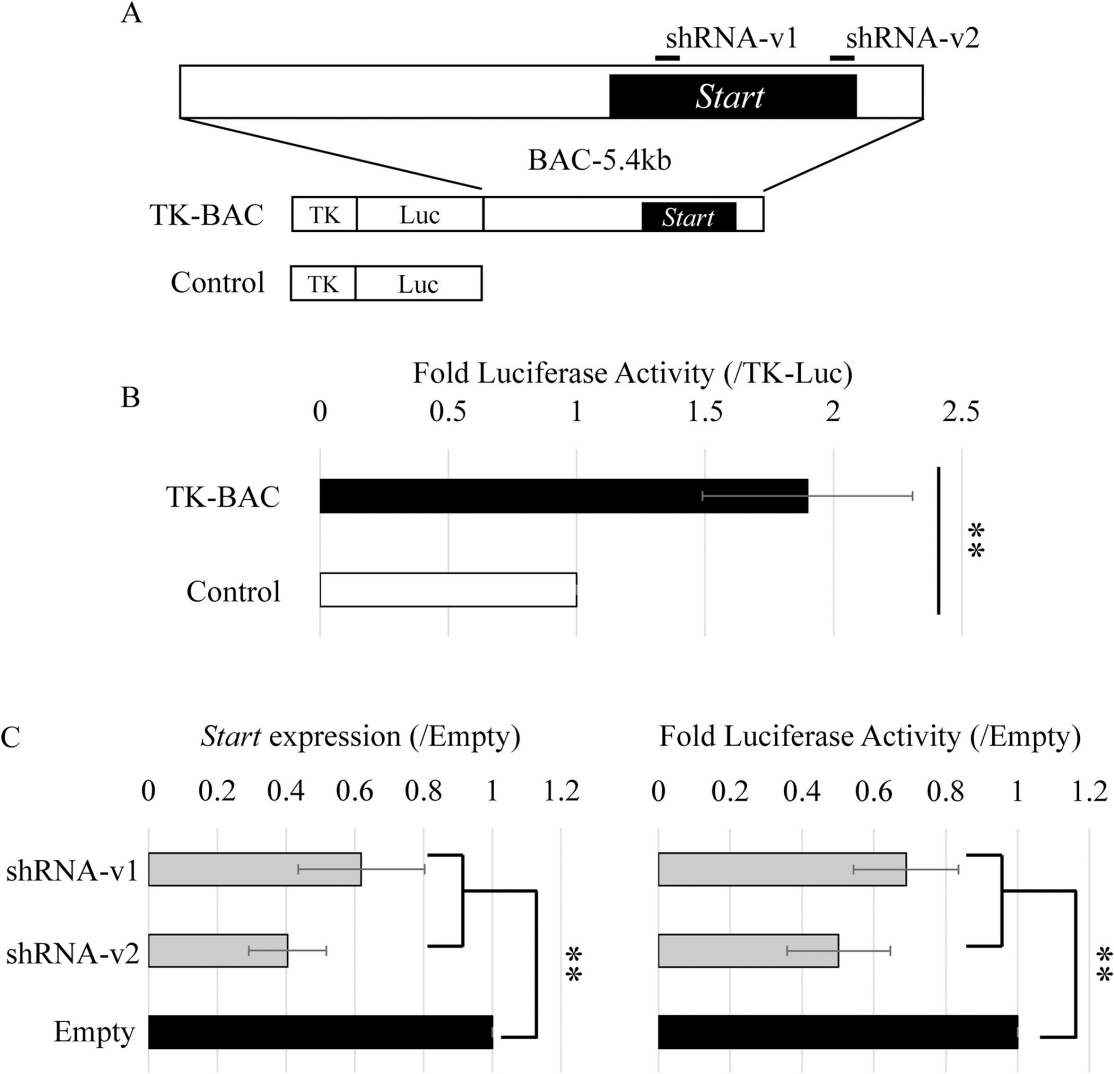
Effects of knockdown (KD) on enhancer activity of the BAC-5.4kb genome sequence encompassing *Start*. (A) Reporter constructs and positions of sequences targeted by shRNAs. The BAC-5.4kb sequence shown at the top is derived from a BAC clone and carries the full length of *Start* (a black box). Two short lines represent sequences targeted by shRNAs for the knockdown (KD) experiment in (C). The “Control” vector contains the firefly luciferase gene (Luc) driven by the TK promoter. The “TK-BAC” construct has the BAC-5.4kb sequence downstream of Luc of the “Control” vector. (B) Enhancer activity of the BAC-5.4kb by a reporter gene assay. The “TK-BAC” and “Control” vectors were transfected into GC-2spd(ts) cells, and two days later, the cells were collected, and firefly luciferase activity was measured. Each value was normalized to that of *Renilla* luciferase activity, which was derived from a co-transfected construct. Data are presented as means ± S.D. from six independent experiments. Statistical significance was analyzed by Student’s t-test. **P < 0.01. (C) KD of *Start* decreased enhancer activity of TK-BAC-5.4kb. (Left) KD efficiency of two shRNAs (shRNA-v1 and shRNA-v2) was evaluated by qRT-PCR. The “TK-BAC” construct was stably transfected into GC-2spd(ts) cells, and two shRNA constructs and a control construct (Empty) were transiently transfected into these stable cell lines. After selection with hygromycin, total RNA was collected from each sample and used for qRT-PCR. The *Gapdh* gene was used as an internal control to normalize the expression level of each gene. (Right) The effect of *Start*-KD on enhancer activity of BAC-5.4kb was evaluated by a reporter gene assay. Firefly luciferase activity was measured and normalized to the amount of protein determined by the BCA assay. In both graphs, the value in the “Empty” group was set to 1.0. Data are presented as means ± S.D. from five independent experiments. Statistical significance was analyzed by one-way ANOVA followed by Dunnett’s test. **P < 0.01.

To examine whether *Start* possessed transcriptional regulatory activity, we performed a reporter gene assay using a 5.4-kb genome sequence and the TK promoter (Fig 2A). This 5.4-kb sequence encompassed the full length of *Start* (1822 bp) and its 2886-bp upstream and 702-bp downstream sequences, and the TK promoter was used as a promoter to drive the luciferase gene. As a model for this reporter gene assay, we used mouse spermatocyte-derived GC-2spd(ts) cells. Although this cell line is one of good models for the study of testicular germ cells, it is different from actual spermatocytes in terms of its phenotype and gene expression pattern [34, 35] Thus, the results should be interpreted with caution.

In GC-2spd(ts) cells, the 5.4-kb sequence significantly elevated TK promoter activity (Fig 2B), and in this assay, expression of the *Start* transcript was detected only in the cells with TK-BAC (S2 Fig). To examine whether the *Start* transcript contributed to enhancer activity of the 5.4-kb sequence, we established GC-2spd(ts) cells stably transfected with TK-BAC and performed *Start* KD. Two shRNAs decreased *Start* expression by approximately 40% and 60%, respectively, which led to a significant decrease in luciferase activity compared to the control (Fig 2C). These results supported the notion that *Start* contributed to the enhancer activity of the 5.4-kb sequence for the TK promoter.

**Fig 3:**
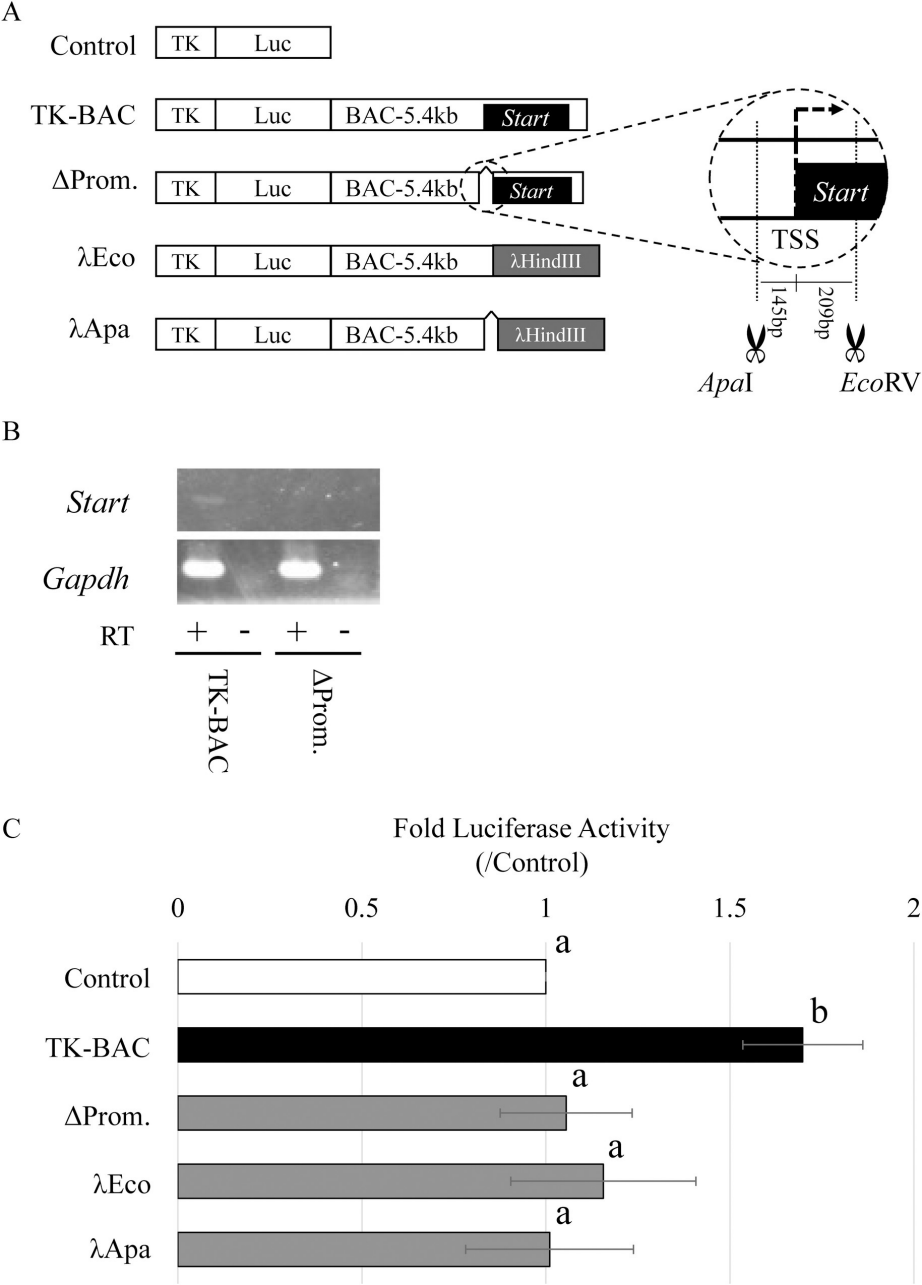
Attenuated enhancer activity caused by deletion of the *Start* promoter in the BAC-5.4kb sequence. (A) Reporter constructs used in (B) and (C). “Control” and “TK-BAC” were described in the legend of Fig 2A. The “ΔProm” construct lacked a 354-bp sequence around the transcriptional start site of *Start* in “BAC-5.4kb”. This deletion is shown in detail in an enlarged circle on the right. The “λEco” construct carries a 2.3-kb λHindIII fragment in replacement with the *Start* sequence in “BAC-5.4kb”. The “λApa” construct lacks a 354-bp sequence around the transcriptional start site of *Start* in “λEco”. (B) Expression of *Start* in GC-2spd(ts) cells transfected with TK-BAC and ΔProm. Total RNA was collected from each sample and used for RT-PCR. The *Gapdh* gene was used as a positive control. Reverse transcription was done with (+) or without reverse transcriptase (-). The cycle numbers of PCR were 30 for *Start* and 25 for *Gapdh*. (C) Loss of enhancer activity in the BAC5.4-kb sequence by a deletion of the *Start* promoter and a replacement of the *Start* sequence. Each construct in (A) was separately transfected into GC-2spd(ts) cells. Two days later, firefly luciferase activity was measured, and each value was normalized to *Renilla* luciferase activity. The value in “Control” was set to 1.0. Data are presented as means ± S.D. from six independent experiments. Statistical significance was analyzed by one-way ANOVA followed by Dunnett’s test. Different letters represent the statistical significance (*P* < 0.01) among experimental groups.

**Fig 4:**
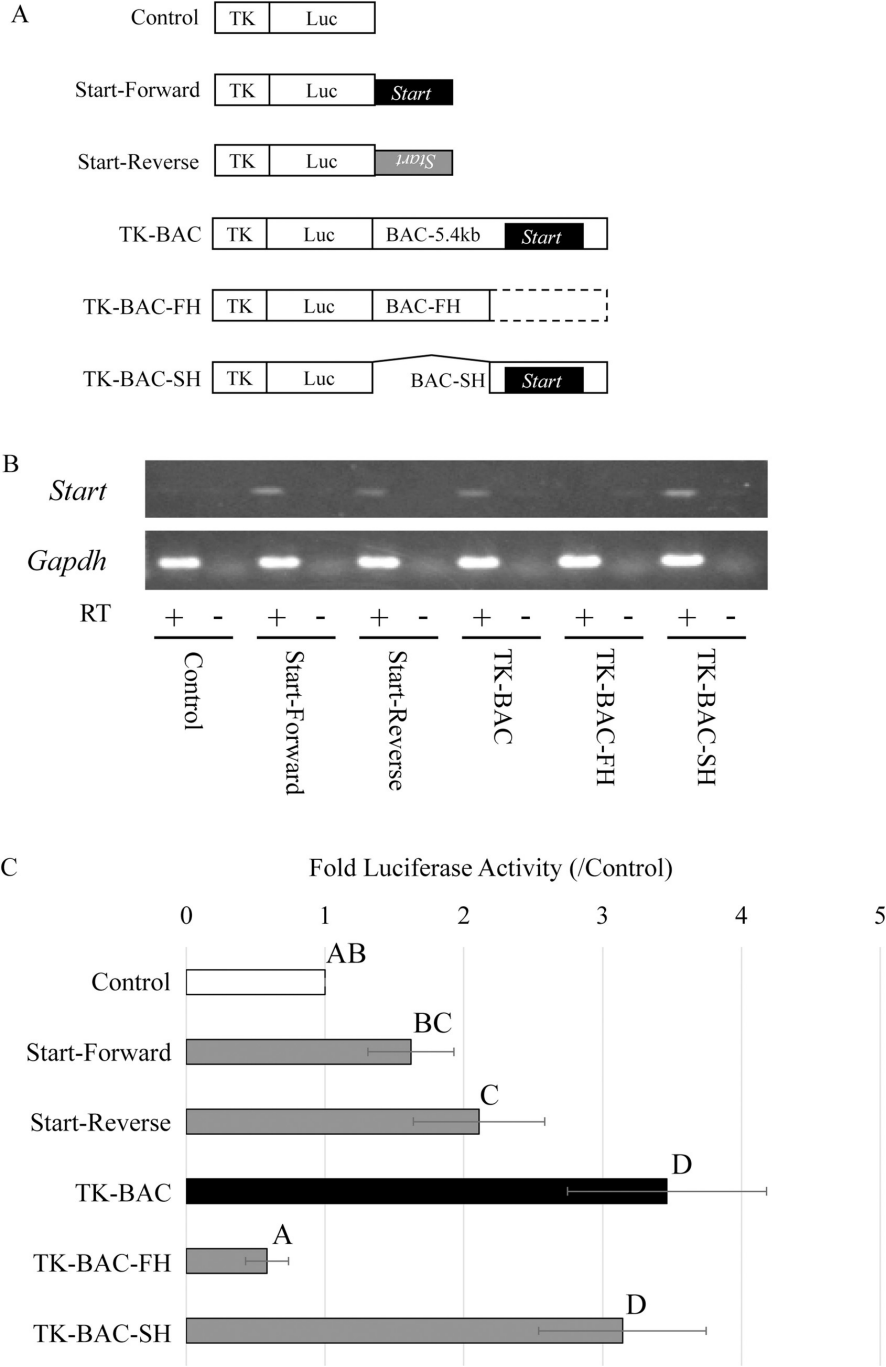
Enhancer activity of parts of the BAC-5.4kb sequence. (A) Reporter constructs used in (B) and (C). “Control” and “TK-BAC” were described in the legend of Fig 2A. The “Start-Forward” and “Start-Reverse” constructs contained the *Start* sequence downstream of the firefly luciferase gene (Luc) driven by the TK promoter in the forward and reverse directions, respectively. The “BAC-FH” and “BAC-SH” constructs contained the TK-driven Luc connected to the first half and the second half of the BAC-5.4kb genome sequence, respectively. (B) *Start* transcription from each construct in a reporter gene assay. Total RNA was collected from each sample and used for RT-PCR. The *Gapdh* gene was used as a positive control. Reverse transcription was done with (+) or without reverse transcriptase (-). The cycle numbers of PCR were 30 for *Start* and 25 for *Gapdh*. (C) Enhancer activity in each part of the BAC-5.4kb sequence encompassing *Start*. The constructs shown in (A) were separately transfected into GC-2spd(ts) cells. Two days later, firefly luciferase activity was measured and normalized to *Renilla* luciferase activity, which was derived from a co-transfected construct. The value in “Control” was set to 1.0. Data are presented as means ± S.D. from nine independent experiments. Statistical significance was analyzed by one-way ANOVA followed by post-hoc Tukey HSD test. Different letters represent the statistical significance (*P* < 0.01) among experimental groups.

To further assess the activity of *Start*, we performed additional reporter gene assays in GC-2spd(ts) cells. First, we deleted the *Start* promoter and replaced the *Start* sequence with λ-DNA in the 5.4-kb sequence (Fig 3A). The promoter deletion, which greatly reduced *Start* expression (Fig 3B), abolished the enhancer activity, and the construct with the replacement did not show enhancer activity (Fig 3C). Second, to check the enhancer activity of each part of the 5.4-kb sequence, we prepared constructs containing the *Start* sequence alone and the first and second halves of the 5.4-kb sequence (Fig 4A). *Start* expression was detected in the cells with Start-Forward, Start-Reverse, TK-BAC and TK-BAC-SH (Fig 4B), and luciferase activity was higher in these constructs than in Control (Fig 4C). These findings indicate that the 5.4-kb genome sequence shows enhancer activity for the TK promoter when *Start* is actively transcribed and that the *Start* transcript contributes to the enhancer activity.

### Activation of a *Prss43/Tessp-3* promoter by the *Start* transcript

**Fig 5:**
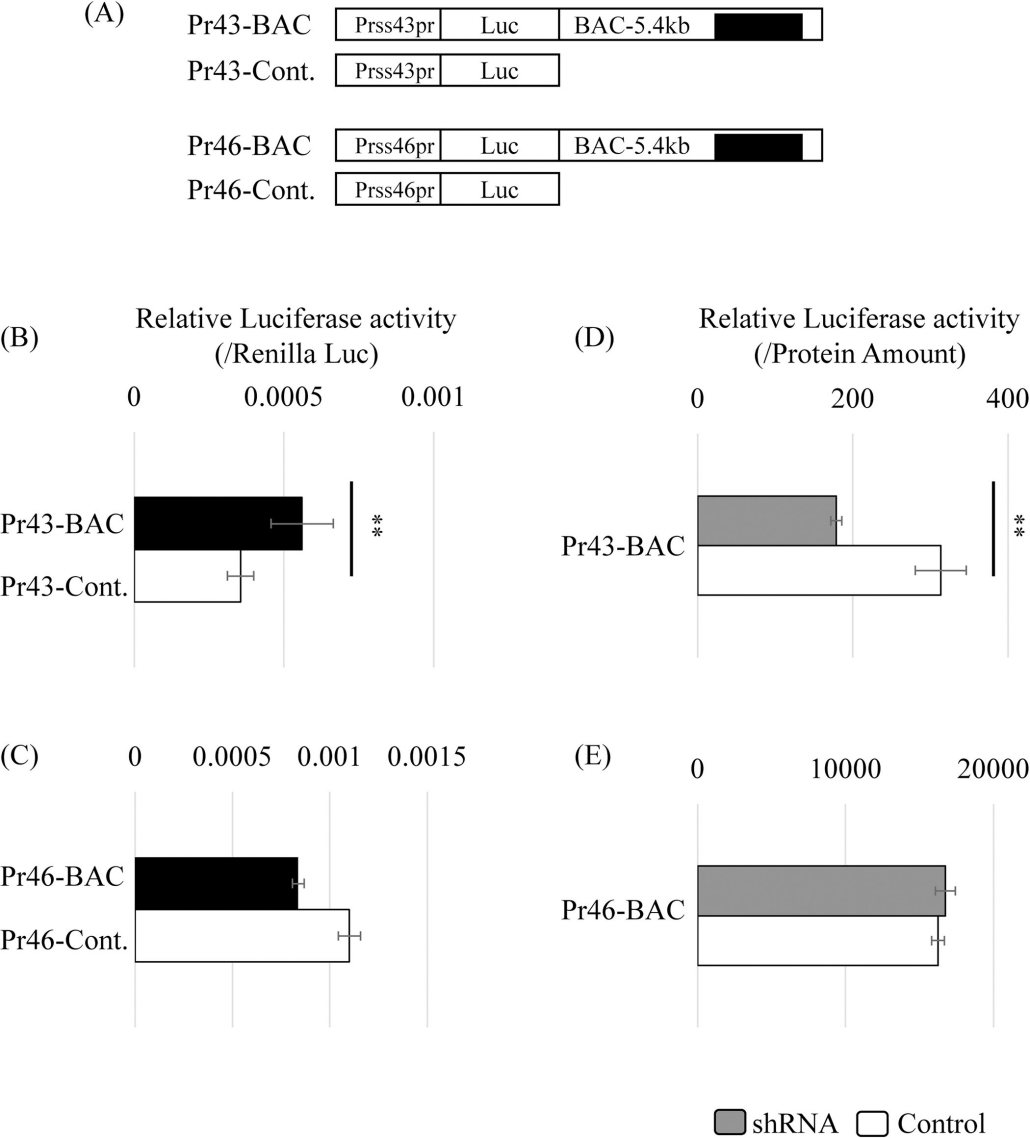
The BAC-5.4kb sequence increased promoter activity of *Prss43/Tessp-3* gene. (A) Reporter constructs used in (B)-(E). The promoter sequence of each *Prss/Tessp* gene was inserted at the upstream of the firefly luciferase gene which was connected to the BAC-5.4kb sequence encompassing *Start*. (B, C) Enhancer activity of the BAC-5.4kb sequence for *Prss43/Tessp-3* and *Prss46* promoters by a reporter assay. Each construct depicted in (A) was transfected into GC-2spd(ts) cells, and two days later, firefly luciferase activity was measured. Each value was normalized to that of *Renilla* luciferase activity, which was derived from a co-transfected construct. Data are presented as means ± S.D. from three independent experiments. Statistical significance was analyzed by Student’s t-test. **P < 0.01. (D, E) Effects of *Start*-KD on enhancer activity of the 5.4-kb sequence. Each construct containing BAC-5.4kb in (A) was stably transfected into GC-2spd(ts) cells. The shRNA-v2 for *Start* was then transfected into the established cells, and after selection with hygromycin, firefly luciferase activity was measured. Each value was normalized to the amount of protein determined by the BCA assay. Data are presented as means ± S.D. from three independent experiments. Statistical significance was analyzed by Student’s t-test. **P < 0.01.

We then investigated the contribution of *Start* to the transcriptional activation of the *Prss43/Tessp-3* gene. The 5.4-kb sequence was connected to the luciferase gene driven by the *Prss43/Tessp-3* promoter and the *Prss46* promoter as a control (Fig 5A). A reporter gene assay in GC-2spd(ts) cells showed that the 5.4-kb sequence exhibited significant enhancer activity for the *Prss43/Tessp-3* promoter but not for the *Prss46* promoter (Fig 5B and 5C). To examine the contribution of the *Start* transcript to the enhancer activity, we established GC-2spd(ts) cells stably transfected with Pr43-BAC and Pr46-BAC, and shRNA-v2 for *Start* was transfected. As expected, the luciferase activity of Pr43-BAC was significantly decreased by *Start* KD, but that of Pr46-BAC was unchanged (Fig 5D and 5E). These findings suggest that the *Start* transcript functions in the activation of the *Prss43/Tessp-3* gene.

## Discussion

In this study, we assessed transcriptional regulatory activity of *Start* by using the 5.4-kb BAC sequence and showed evidence for its role in activation of the TK promoter by a series of reporter gene assays. First, the enhancer activity of the 5.4-kb sequence was decreased by *Start* KD (Fig 2). Second, deletion of the *Start* promoter (ΔProm) greatly impaired *Start* transcription and diminished the enhancer activity (Fig 3). Third, replacement of the *Start* sequence with λ-DNA (λEco) decreased the enhancer activity (Fig 3). Fourth, partial sequences of the 5.4-kb BAC that drove *Start* expression showed higher enhancer activity than that of sequences not driving *Start* transcription (Fig 4). These findings indicate that *Start* transcription plays a role in activation of the TK promoter. Considering significant decreases of the activity by *Start* KD, *Start* is presumed to function in transcriptional activation as a transcript.

Since lncRNAs are frequently associated with enhancers [36, 37], whether the 5.4-kb genome sequence itself possesses enhancer activity is an important question. To answer this, constructs that do not drive *Start* transcription are useful. The ΔProm construct, which contained most of the 5.4-kb sequence but did not drive *Start* transcription, showed no enhancer activity (Fig 3). The deleted sequence in ΔProm remained in the λEco construct, which also showed no enhancer activity. Therefore, it is assumed that the 5.4-kb genome sequence does not possess enhancer activity. However, it is also true that TK-BAC and TK-BAC-SH showed higher activity than Start-Forward and Start-Reverse (Fig 4), which suggests that some sequences surrounding *Start* contribute to increasing the enhancer activity. It is possible that both transcript and genomic sequence contribute to the enhancer activity, and our current findings strongly suggest that the *Start* transcript functions in transcriptional activation. Given that the *Start* transcript is localized in the nucleus of pachytene spermatocytes, *Start* is presumed to play a role in transcriptional activation at this stage.

As a potential target of *Start*, we investigated the *Prss43/Tessp-3* gene because it was greatly downregulated in *Start*-KO testes (Fig 1). The enhancer activity of the 5.4-kb sequence for a *Prss43/Tessp-3* promoter was significantly decreased by *Start* KD (Fig 5), suggesting that the *Start* transcript from the 5.4-kb sequence functioned in transcriptional activation of *Prss43/Tessp-3*. Moreover, *Start* expression is elevated at 14 days postpartum, a few days earlier than *Prss43/Tessp-3* activation during testis development [27, 30]. Therefore, the *Prss43/Tessp-3* gene is likely to be a target of *Start*. Because the expression was decreased by 92% in *Start*-KO testes (Fig 1C), *Start* is probably a critical factor for *Prss43/Tessp-3* gene activation.

It is interesting that *Start*-KO mice showed normal spermatogenesis despite a substantial decrease in expression of the *Prss43/Tessp-3* gene, which is considered to be important for the progression of meiosis [27]. This may be because the low level of expression in *Start*-KO testes was sufficient for meiosis or because other proteases compensated the function of *Prss43/Tessp-3*, as has been suggested for some testicular genes [38, 39]. Alternatively, abnormality may appear at younger or older ages than the ages of mice examined in this study. The phenotype of *Start*-KO mice is being investigated in more detail.

It is also remarkable that *Start* transcription was detected from Start-Forward and Start-Reverse constructs that included the entire *Start* sequence and no neighboring sequences (Fig 4). This suggests that the *Start* full-length sequence contained a minimum promoter. Given that the deletion of a 354-bp sequence around the transcriptional start site of *Start* abolished the *Start* transcription (Fig 3), the 209-bp region of the 5’ portion of the *Start* sequence might function as a minimum promoter. Elucidation of the detailed mechanism by which *Start* transcription is regulated will be one of our future challenges.

How could *Start* have different function in different cell types by targeting different genes? A clue to answering this question is the subcellular localization. An lncRNA could regulate transcription or could be a component of subnuclear structure in the nucleus [1, 2], whereas a cytosolic lncRNA could regulate gene expression by affecting the mRNA stability or controlling translation [40, 41]. Moreover, co-regulators are different between the nucleus and cytosol. While nuclear lncRNAs bind to transcription factors or epigenetic factors, cytosolic lncRNAs interact with miRNAs [42–46]. Thus, the identification of binding partners with *Start* in the nucleus of spermatocytes and in cytosol of Leydig cells will be required to understand more detailed mechanisms of gene regulation by *Start*. Such studies are ongoing.

Only a few of the many lncRNAs expressed in the testis have been functionally assessed and characterized [47–50]. Some mouse models with KO of lncRNAs in germ cells showed a defect in meiosis, smaller number of sperm, and less motile sperm, although no phenotype was reported in other KO mice [8–10, 51]. In Leydig cells, lncRNAs were predicted to regulate the miRNA-mRNA network [20, 52–55]. lncRNAs in either germ cells or Leydig cells were investigated in these studies. In this study, we found that *Start*, an lncRNA that regulates steroidogenesis in Leydig cells probably through a mechanism in the cytosol, is involved in transcriptional activation of a protease gene in the nucleus of germ cells. To the best of our knowledge, this is the first example of a multifunctional lncRNA in the testis. Since several lncRNAs were reported to be expressed in different cell types in the testis, with different subcellular localizations, or in different tissues including the testis [28, 29, 52, 56], more testicular lncRNAs may be multifunctional. Our findings provide an insight into a further understanding of the multifunctionality of testis lncRNAs.

## Supporting information

S1 Fig Plasmid construction. (A) Construction of “ΔProm”. (B) Construction of “λEco”. (C) Construction of “λApa”. (D) Construction of “TK-BAC-SH”. (E) Construction of “TK-BAC-SH”.
(PDF)

S2 Fig Expression of *Start* in GC-2spd(ts) cells transfected with TK-BAC and control. Total RNA was collected from each sample and used for RT-PCR. The *Gapdh* gene was used as a positive control. Reverse transcription was done with (+) or without reverse transcriptase (-). The cycle numbers of PCR were 35 for *Start* and 25 for *Gapdh*, respectively.
(PDF)

S1 Data (XLSX)

S1 Raw images (PDF)
