# Supplementary material for: Evidence for a functional role of Start, a long noncoding RNA, in mouse spermatocytes
Source: PLoS One. 2022 Aug 25;17(8):e0273279. doi: 10.1371/journal.pone.0273279 (PMC9409574; doi:10.1371/journal.pone.0273279)
Supplement: S1 Fig — (A) Construction of “ΔProm”. (B) Construction of “λEco”. (C) Construction of “λApa”. (D) Construction of “TK-BAC-SH”. (E) Construction of “TK-BAC-SH”. (PDF) [file pone.0273279.s001.pdf]

“ΔProm”

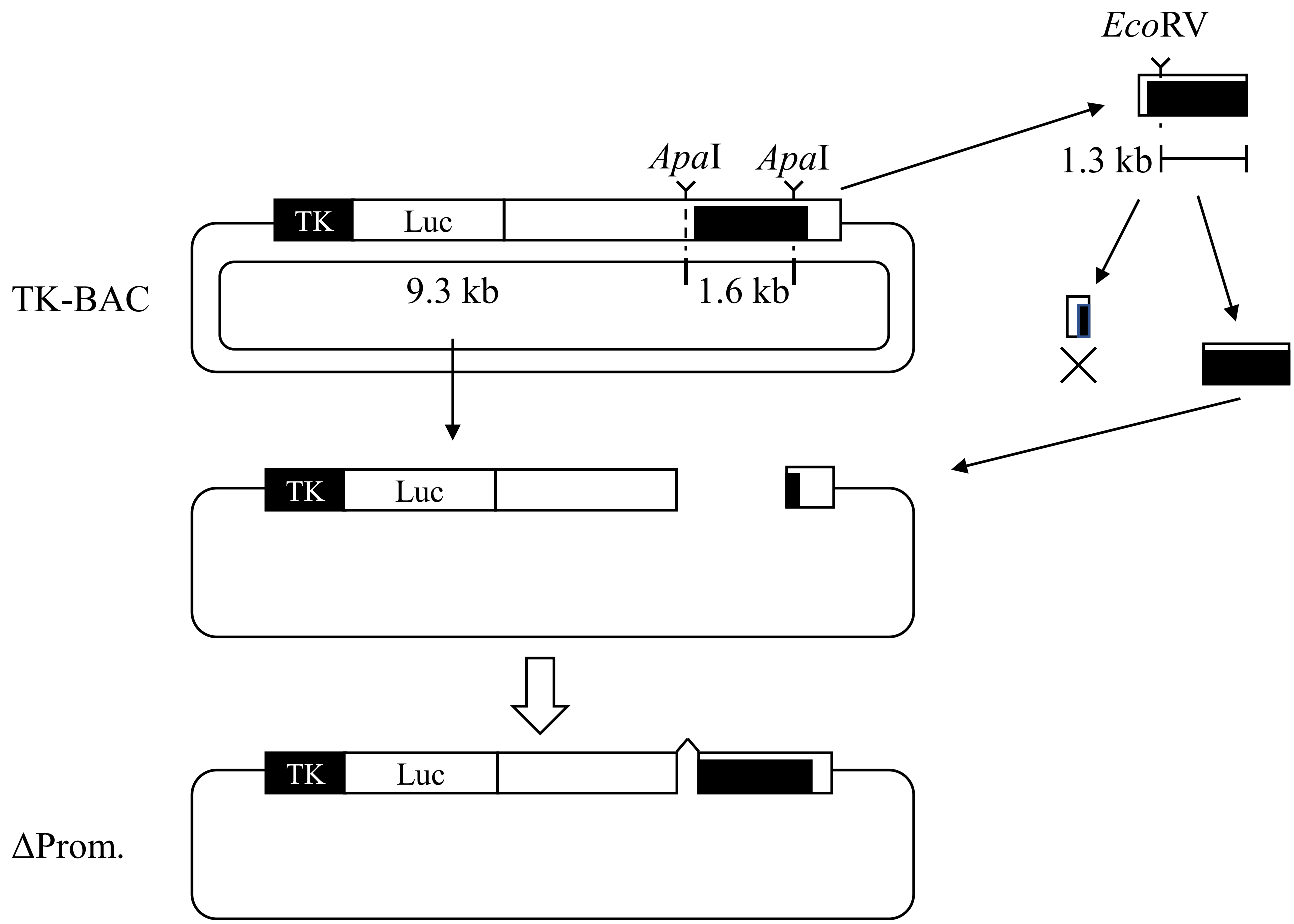

S1A Fig. Construction of “ΔProm”.

“λEco”

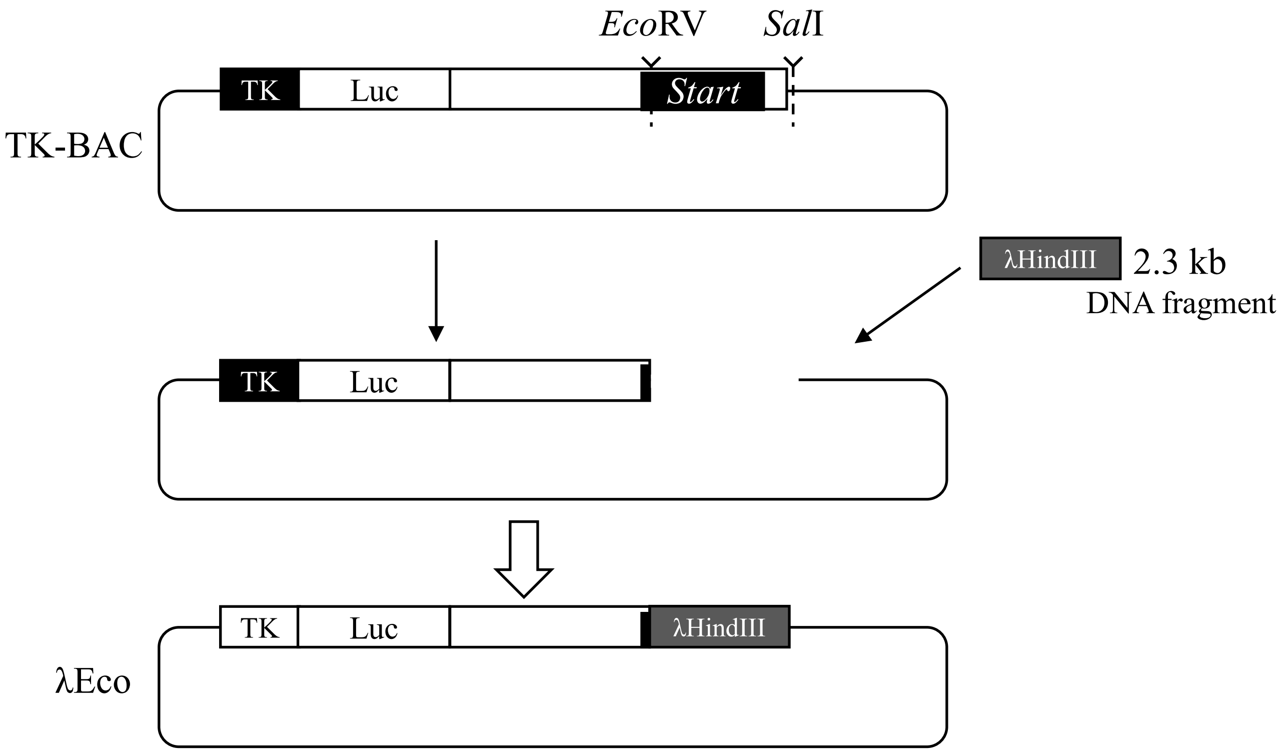

S1B Fig. Construction of “λEco”.

“λApa”

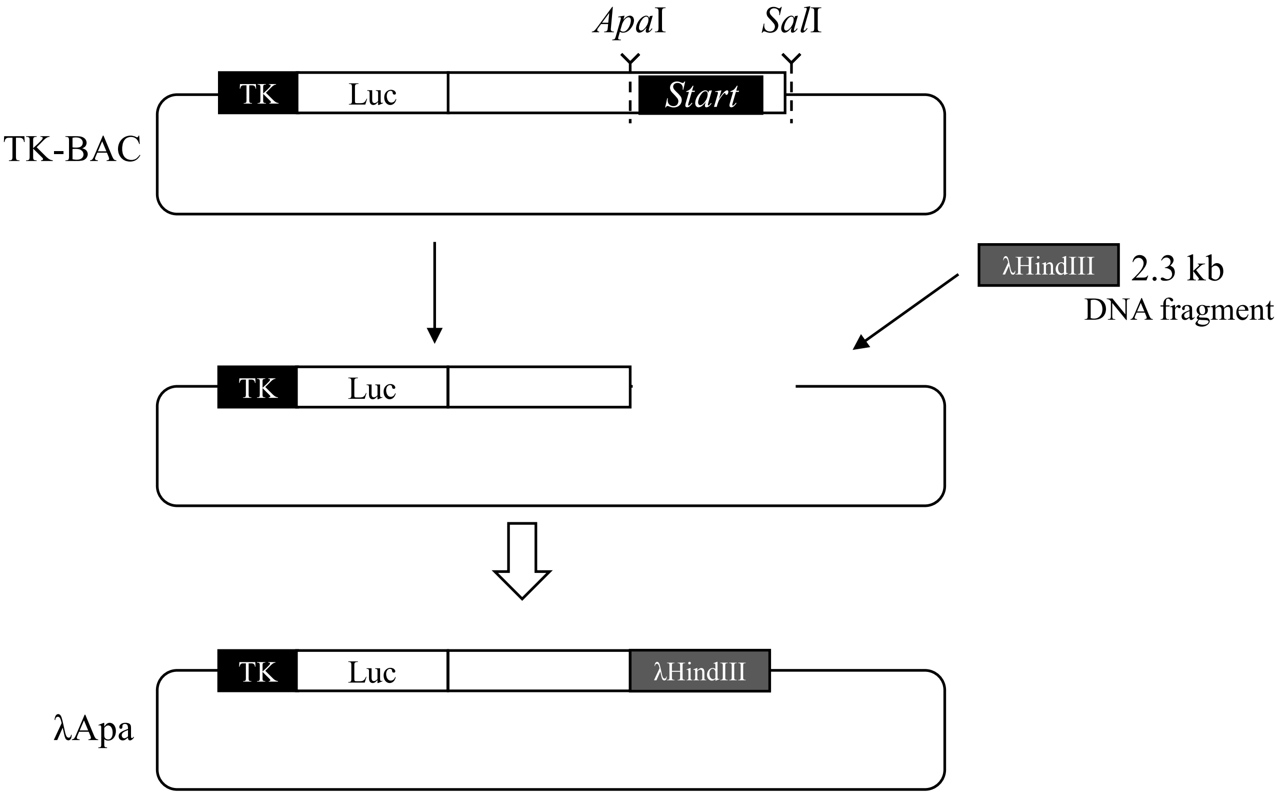

S1C Fig. Construction of “λApa”.

"TK-BAC-SH"

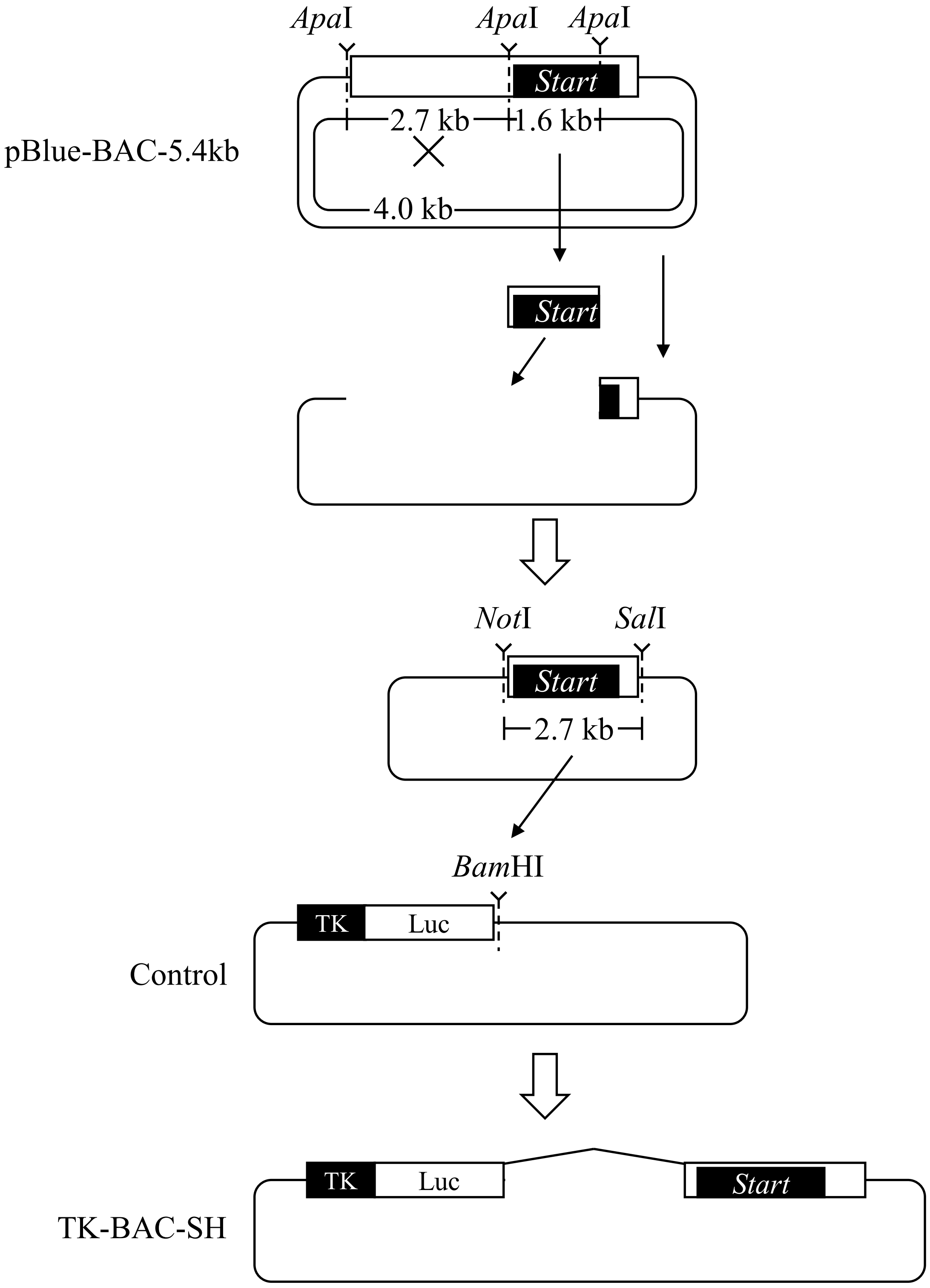

S1D Fig. Construction of "TK-BAC-SH".

“TK-BAC-FH”

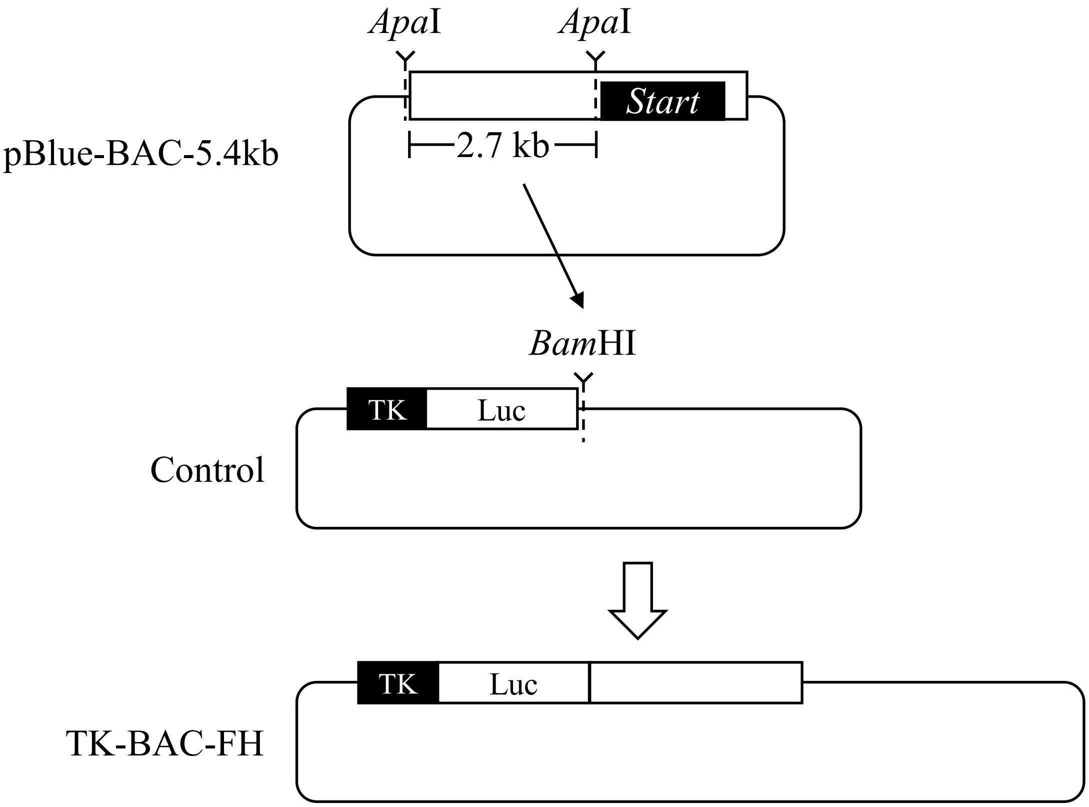

S1E Fig. Construction of “TK-BAC-SH”.
