# Supplementary material for: Evidence for a functional role of Start, a long noncoding RNA, in mouse spermatocytes
Source: PLoS One. 2022 Aug 25;17(8):e0273279. doi: 10.1371/journal.pone.0273279 (PMC9409574; doi:10.1371/journal.pone.0273279)
Supplement: S2 Fig — Total RNA was collected from each sample and used for RT-PCR. The Gapdh gene was used as a positive control. Reverse transcription was done with (+) or without reverse transcriptase (-). The cycle numbers of PCR were 35 for Start and 25 for Gapdh, respectively. (PDF) [file pone.0273279.s002.pdf]

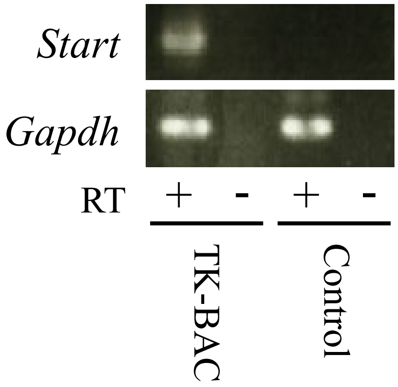

S2 Fig. Expression of *Start* in GC-2spd(ts) cells transfected with TK-BAC and Control. Total RNA was collected from each sample and used for RT-PCR. The *Gapdh* gene was used as a positive control. Reverse transcription was done with (+) or without reverse transcriptase (-). The cycle numbers of PCR were 35 for *Start* and 25 for *Gapdh*, respectively.
