# Supplementary figures and images for: Evidence for a functional role of Start, a long noncoding RNA, in mouse spermatocytes
Source: PLoS One. 2022 Aug 25;17(8):e0273279. doi: 10.1371/journal.pone.0273279 (PMC9409574; doi:10.1371/journal.pone.0273279)

Gel images for Fig 3

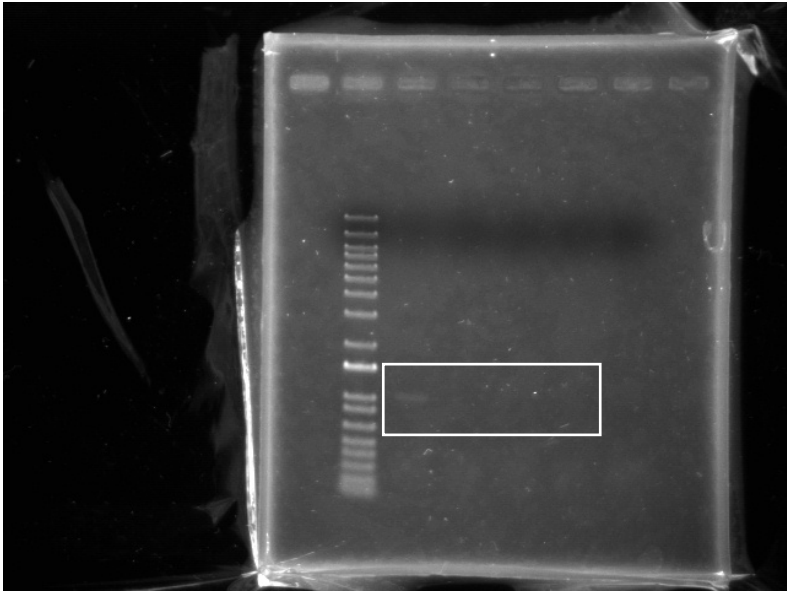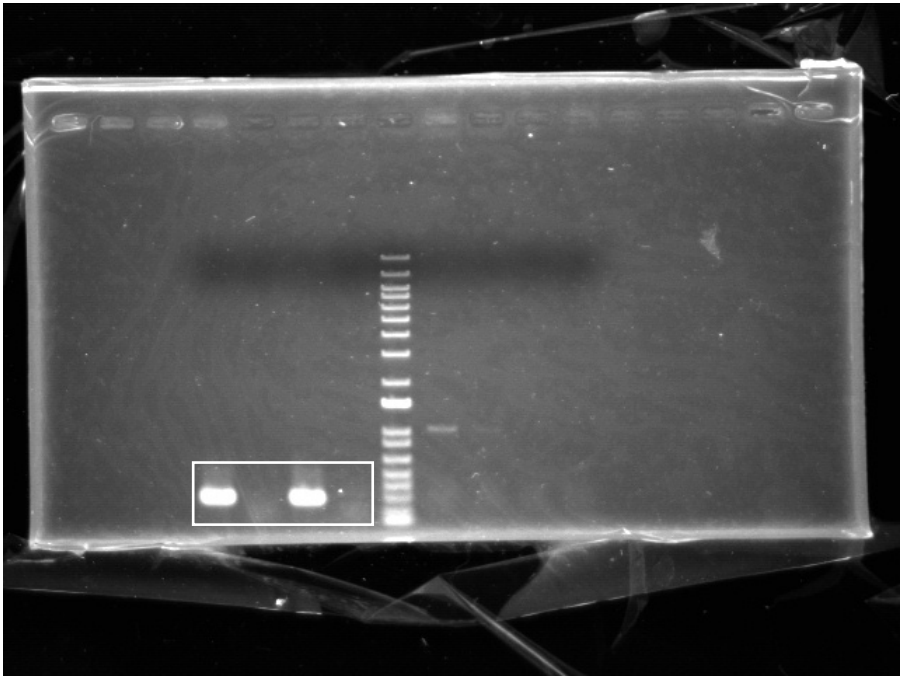

Gel images for Fig 4

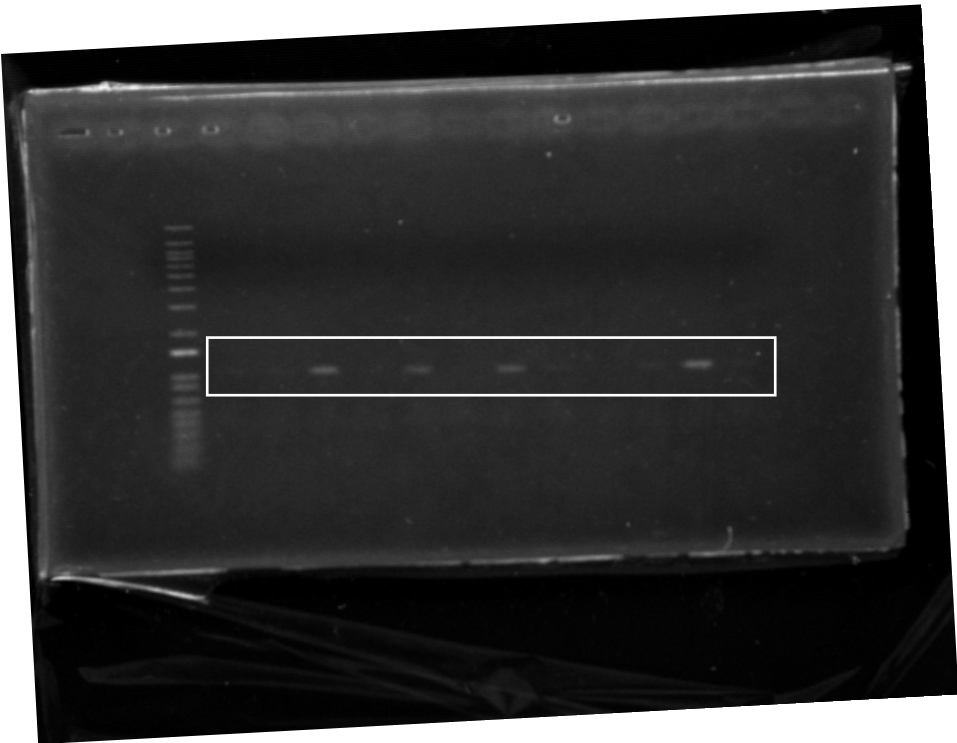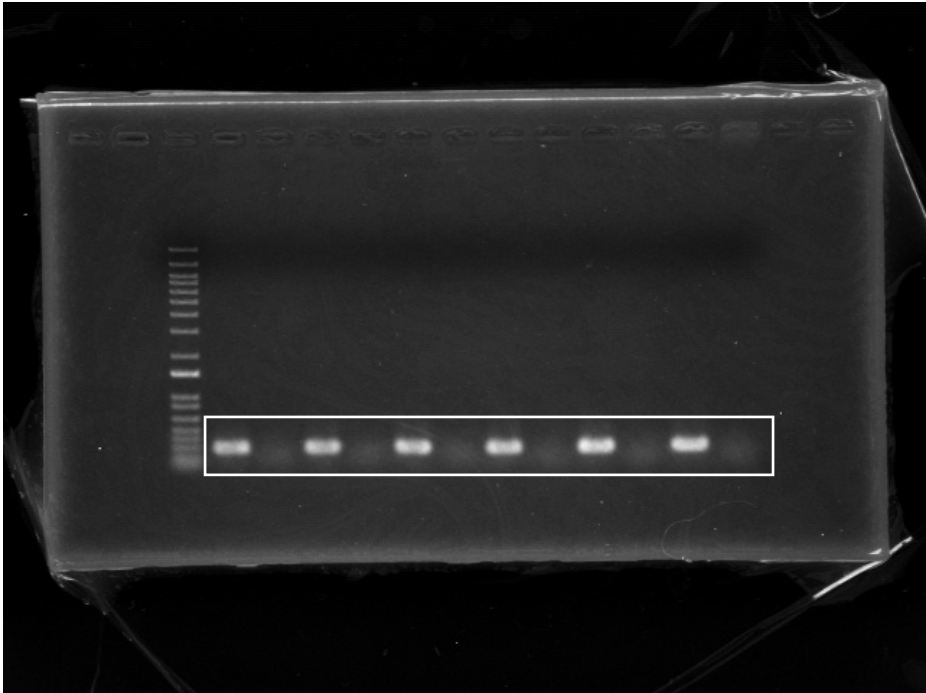

Gel images for S2 Fig

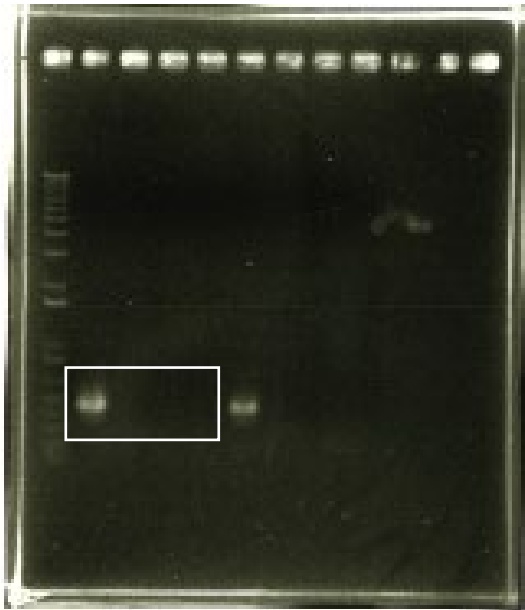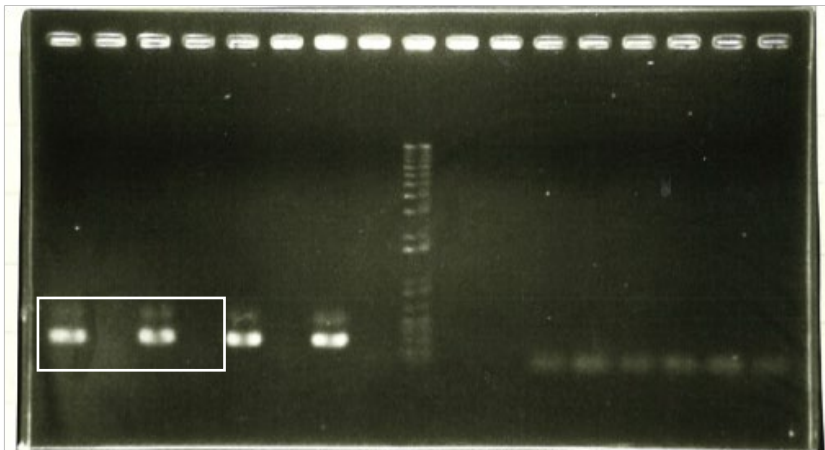

Supplement: S1 Raw images — (PDF) [file pone.0273279.s004.pdf]
